# Hospital days, hospitalization costs, and inpatient mortality among patients with mucormycosis: a retrospective analysis of US hospital discharge data

**DOI:** 10.1186/1471-2334-14-310

**Published:** 2014-06-05

**Authors:** Marya D Zilberberg, Andrew F Shorr, Huan Huang, Paresh Chaudhari, Victoria Federico Paly, Joseph Menzin

**Affiliations:** 1 EviMed Research Group, LLC, Goshen, MA, USA; 2 School of Public Health and Health Sciences, University of Massachusetts Amherst, Amherst, MA, USA; 3 Washington Hospital Center, Washington, DC, USA; 4 Boston Health Economics, Inc., Waltham, MA, USA; 5 Astellas Scientific and Medical Affairs, Inc., Northbrook, IL, USA

**Keywords:** Mucormycosis, Costs, Mortality, Fungal infections

## Abstract

**Background:**

Mucormycosis is a rare and potentially fatal fungal infection occurring primarily in severely immunosuppressed patients. Because it is so rare, reports in the literature are mainly limited to case reports or small case series. The aim of this study was to evaluate inpatient mortality, length of stay (LOS), and costs among a matched sample of high-risk patients with and without mucormycosis in a large nationally representative database.

**Methods:**

We conducted a retrospective analysis using the 2003–2010 Healthcare Cost and Utilization Project – Nationwide Inpatient Sample (HCUP-NIS). The NIS is a nationally representative 20% sample of hospitalizations from acute care United States (US) hospitals, with survey weights available to compute national estimates. We classified hospitalizations into four mutually exclusive risk categories for mucormycosis: A- severely immunocompromised, B- critically ill, C- mildly/moderately immunocompromised, D- major surgery or pneumonia. Mucormycosis hospitalizations (“cases”) were identified by ICD-9-CM code 117.7. Non-mucormycosis hospitalizations (“non-cases”) were propensity-score matched to cases 3:1. We examined demographics, clinical characteristics, and hospital outcomes (mortality, LOS, costs). Weighted results were reported.

**Results:**

From 319,366,817 total hospitalizations, 5,346 cases were matched to 15,999 non-cases. Cases and non-cases did not differ significantly in age (49.6 vs. 49.7 years), female sex (40.5% vs. 41.0%), White race (53.3% vs. 55.9%) or high-risk group (A-49.1% vs. 49.0%, B-20.0% vs. 21.8%, C-25.5% vs. 23.8%, D-5.5% vs. 5.4%). Cases experienced significantly higher mortality (22.1% vs. 4.4%, P < 0.001), with mean LOS and total costs more than 3-fold higher (24.5 vs. 8.0 days and $90,272 vs. $25,746; both P < 0.001).

**Conclusions:**

In a national hospital database, hospitalizations with mucormycosis had significantly higher inpatient mortality, LOS, and hospital costs than matched hospitalizations without mucormycosis. Findings suggest that interventions to prevent or more effectively treat mucormycosis are needed.

## Background

Mucormycosis (formerly zygomycosis) is a rare invasive fungal infection (IFI) associated with substantial morbidity and mortality. Immunosuppression is a common predisposing factor for mucormycosis, with the infection generally limited to patients with hematological malignancies or hematopoietic stem cell transplants, solid-organ transplants, and diabetes [1-5]. A review of 929 mucormycosis cases reported since 1940 found the most common underlying conditions/risk factors to be diabetes (36%), malignancies (17%), and organ transplant (bone marrow or solid organ; 12%) [5]. Between 2001 and 2005, analyses of data from the Transplant Associated Infections Surveillance Network (TRANSNET), showed that mucormycosis represented 8% and 2% of IFIs found in patients receiving hematopoietic stem cell and solid organ transplant recipients, respectively [6,7]. More recent publications from Europe and Asia highlight the increasing recognition of mucormycosis around the globe, and also present data supporting a shift from such traditional risk factors, with hematological malignancies as the most common underlying condition [8-10].

With advances in treatment, mortality rates among patients infected with mucormycosis have declined [3], but recent estimates of 90-day mortality continue to range from 20% to 58% [1-3,11,12]. Hospitalizations for mucormycosis are also associated with prolonged hospital length of stay, substantial use of intensive care services, and excess costs of over $30,000 [13-15].

To date, mucormycosis has been studied in small and narrowly defined populations or as a subgroup within a larger sample of patients with IFIs, resulting in sample sizes of 200 cases or fewer [2-4,11-13,15]. Such studies have limited utility in understanding the true clinical and economic burden of this condition. To address these gaps, we evaluated the epidemiology, inpatient mortality, hospital days, and hospitalization costs among a large sample of hospitalized patients with mucormycosis using a United States (US) nationally representative inpatient dataset.

## Methods

### Data source

We conducted a cost-of-illness analysis of hospital discharge data from the Healthcare Cost and Utilization Project—Nationwide Inpatient Sample (HCUP-NIS) from 2003 through 2010. The HCUP-NIS, maintained by the Agency for Healthcare Research and Quality (AHRQ), is a 20% stratified representative sample of all US inpatient stays in acute-care non-federally funded institutions. It contains records on approximately 8 million hospitalizations each year from over 1000 US facilities.

Core hospital stay files contain details on patient demographics (e.g., age, sex, race), International Classification of Diseases, 9th Revision, Clinical Modification (ICD-9-CM) diagnosis codes (15–25, depending on the year), Elixhauser comorbidities [16], length of hospital stay, discharge status, and total charges. Each hospitalization is assigned a specific sample weight used to estimate national rates. Hospital characteristics, such as geographic location, teaching status, number of beds, and hospital-specific cost-to-charge ratios are provided in separate files and can be linked to the hospitalization records.

The HCUP-NIS data used in this study represent de-identified human subject data. The database does not contain data elements that would allow direct or indirect identification of specific individuals. All parties with access to the data were signatories of HCUP’s formal data use agreement (DUA), including the provision that no cell sizes less than 10 can be reported, and additionally completed the HCUP DUA Training. This provision is deemed by AHRQ to be an adequate safeguard against identification of individual patients.

### Sample selection

Hospitalizations of patients considered to be at risk for mucormycosis were identified based on the following hierarchy of high-risk categories for this infection:

A. Severely immunocompromised (leukemia/lymphoma, hematopoietic stem cell or bone marrow transplant, chemotherapy, other immune disorders, solid organ transplant, long-term steroid use)

B. Critically ill (trauma, septicemia, bacteremia, necrotizing fasciitis, intracranial hemorrhage, mechanical ventilation for over 96 hours)

C. Mildly/moderately immunocompromised (end**-**stage renal disease/hemodialysis, lupus, malignant solid tumors, diabetic ketoacidosis, blood disorders, diabetes)

D. Major surgery or pneumonia

Categories were identified based on ICD-9-CM diagnosis and procedure codes, as well as diagnosis-related group (DRG) codes listed for the admission. Codes by date or time within the hospitalization are not available in the NIS database. Hospitalizations not falling into one of these categories were excluded from the analysis. Because the assignment to a risk group was hierarchical (that is, a hospitalization was only assigned to a lower risk group if it did not meet the criteria for the group above it in the hierarchy), the four groups were mutually exclusive. The categories were developed based on both the distribution of the observed comorbid conditions among all mucormycosis hospitalizations in a preliminary analysis and on the clinical understanding of traditional high risk factors (categories A and C).

All four categories together comprised the analysis pool for hospitalizations at risk for mucormycosis. Among these high-risk groups, hospitalizations with mucormycosis were identified by the ICD-9-CM diagnosis code 117.7 (Zygomycosis [Phycomycosis or Mucormycosis]; cases) and matched to high-risk hospitalizations without a mucormycosis diagnosis (non-cases) based on a propensity score (PS). Since the NIS is strictly an administrative database without any clinical data, we were unable to classify mucormycosis cases further as “proven”, “probable,” or “possible” as recommended by the European Organization for Research and Treatment of Cancer/Invasive Fungal Infections Cooperative Group and the National Institute of Allergy and Infectious Diseases Mycoses Study Group (EORTC/MSG). To minimize center effect, selection of non-cases was restricted to hospitals with at least one mucormycosis hospitalization in the same year.

### Study measures

Patient and hospital characteristics were evaluated for all hospitalizations. We included demographic (age at admission, sex, race, median household income, payer type, and year of admission), clinical (high-risk category, Elixhauser comorbidities [16], number of listed discharge diagnoses, receipt of abdominal procedure/surgery, and total number of listed procedures), hospital (geographic region, urbanicity, teaching status, and size [number of beds]), and admission-specific (source [e.g., emergency room, another hospital, etc.], and urgency [e.g., emergency, urgent, elective]) characteristics.

We examined the following outcomes: in-hospital mortality, discharge destination for survivors (e.g., short-term hospital, skilled nursing facility, intermediate care facility, home health care, etc.), length of stay (LOS), and total hospitalization charges and costs. Costs were derived by applying hospital-specific cost-to-charge ratios provided by HCUP to the charges for each individual hospitalization. All costs and charges were adjusted to 2011 USD using the medical care component of the US Consumer Price Index. Indirect (non-medical) costs were not available in the database and thus excluded from analyses. The NIS data do not allow for the calculation of attributable costs.

### Data analyses

Propensity scores were computed by modeling the likelihood of having mucormycosis using a logistic regression. Predictors included patient (age, sex, race, median household income, payer, number of comorbidities, and high-risk group), hospital (geographic region, urbanicity, teaching status, size), and admission (weekend/weekday admission, presence of abdominal procedure/surgery, and year of admission) characteristics. Predictors were chosen on the basis of an expected or potential relationship to mucormycosis exposure. Data availability was also a factor in selecting predictors. Variables that were not consistently available across years or hospitals were not included to minimize the number of hospitalizations that would need to be excluded.

Non-mucormycosis high-risk hospitalizations from hospitals with at least one mucormycosis hospitalization were matched 3:1 to mucormycosis patients using a greedy 8-to-1 digit matching algorithm [17]. Stratified analyses by the high-risk group were conducted for length of stay, inpatient mortality, and total hospitalization charges and costs.

All study measures were compared between cases and non-cases before and after matching. Mean, standard deviation, median, interquartile range (IQR), minimum and maximum were reported for continuous measures, and proportions were reported for categorical variables. Significance was tested for proportions using the Rao-Scott Chi-Square Test, and for continuous measures using an independent sample t-test. An alpha level of 0.05 was used to assess statistical significance.

## Results

### Patient characteristics

**Figure 1:**
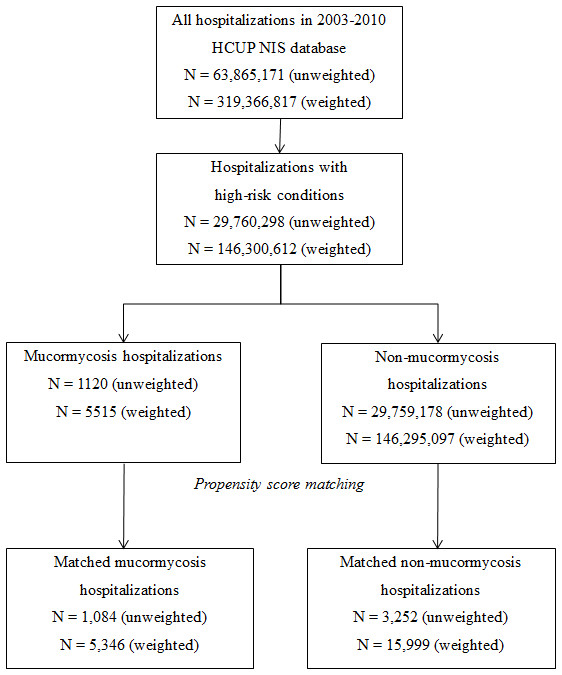
Sample selection flow chart.

Among the total of over 300 million hospitalizations in the US during the study period, 146,300,612 had at least one of the high-risk conditions, of whom 5,515 carried a diagnosis of mucormycosis (< 0.01%, Figure 1). These 5,515 cases represented 94.0% of all hospitalizations that carried a diagnosis of mucormycosis; the remaining 6.0% failed to meet the criteria for a high-risk condition.

Cases differed from non-cases in the unadjusted comparison along most characteristics, including sex, age, race, high-risk group, and number of procedures (Table 1). After propensity score matching 5,346 (96.9%) cases to 15,999 non-cases, many of these differences disappeared. Unless stated otherwise, all the results below represent the post-match data. The mean age for both cases and non-cases was 50 years with the plurality falling into the age group between 45–64 years (43.5% and 43.7% for cases and non-cases, respectively). There were slightly more males than females in both groups (59.5% and 59.0% of cases and non-cases, respectively), and the majority were of White race (53.3% and 55.9%), followed by Hispanic (13.6% and 13.3%) and African American (10.7% and 10.0%). All hospitalizations were evenly distributed across the four median household income quartiles. Private payers were most common in both cases and non-cases (38-40%), followed by Medicare (31-34%) and Medicaid (21%).

**Table 1:** Patient and hospital characteristics

	**Before propensity-score matching**^**1**^			**After propensity-score matching**^**1**^		
**Characteristic**	**Cases**	**Non-Cases**	***P- *** **value**^**3**^	**Cases**	**Non-Cases**	***P- *** **value**^**3**^
N (unweighted)	1,120	29,759,178		1,084	3,252	
N (weighted)	5,515	146,295,097		5,346	15,999	
Sex (%)			< 0.001			0.7550
Female	39.6%	53.2%		40.5%	41.0%	
Male	60.4%	46.6%		59.5%	59.0%	
Unknown	0.0%	0.3%		-	-	
Age (%)						
Mean (SD)	49.5 (20.0)	59.7 (21.4)	< 0.001	49.6 (19.9)	49.7 (19.9)	0.9074
< 44	33.1%	21.4%		33.0%	32.6%	
45 to 64	43.6%	31.0%		43.5%	43.7%	
≥ 65	23.4%	47.6%		23.5%	23.7%	
Race (%)			< 0.001			0.8093
White	53.6%	54.9%		53.3%	55.9%	
African-American	10.5%	10.7%		10.7%	10.0%	
Hispanic	13.5%	7.8%		13.6%	13.3%	
Other/Unknown	22.4%	26.5%		22.4%	20.8%	
Primary expected payer (%)			< 0.001			0.7473
Medicare	33.7%	51.1%		33.6%	31.2%	
Medicaid	21.0%	11.2%		20.8%	20.8%	
Private	37.4%	29.4%		37.8%	39.9%	
Other/Unknown	8.0%	8.3%		7.8%	8.1%	
High-risk conditions^2^ (%)			< 0.001			0.5281
(A) Severely immunocompromised	49.7%	6.2%		49.1%	49.0%	
(C) Mildly/moderately immunocompromised	25.1%	45.5%		25.5%	23.8%	
(B) Critically ill	20.0%	20.3%		20.0%	21.8%	
(D) Major surgery or Pneumonia	5.3%	27.9%		5.5%	5.4%	
Number of Elixhauser comorbidities						
Mean (SD)	2.7 (1.8)	2.4 (1.8)	< 0.001	2.7 (1.8)	2.6 (1.7)	0.0340
Median (IQR)	2 (1–4)	2 (1–3)		3 (1–4)	2 (1–4)	
Number of listed diagnoses						
Mean (SD)	12.1 (4.8)	8.3 (4.3)	< 0.001	12.2 (4.8)	9.2 (4.6)	<0.001
Median (IQR)	11 (9–15)	8 (5–10)		11 (9–15)	9 (6–12)	
Number of listed procedures						
Mean (SD)	5.3 (4.5)	2 (2.3)	< 0.001	5.3 (4.5)	2.5 (2.8)	<0.001
Median (IQR)	5 (2–8)	1 (0–3)		5 (2–8)	2 (1–3)	
Abdominal procedures or surgeries^4^ (%)	5.5%	12.0%	< 0.001	5.4%	4.9%	0.5314
Geographic Region (%)			< 0.001			0.2629
Northeast	15.4%	19.5%		15.4%	16.7%	
Midwest	18.5%	23.6%		18.5%	16.1%	
West	31.9%	18.2%		32.4%	33.3%	
South	34.2%	38.7%		33.6%	33.9%	
Hospital location (%)			< 0.001			0.6274
Urban	95.9%	86.9%		96.8%	97.0%	
Rural	3.1%	12.7%		3.2%	3.0%	
Missing	1.0%	0.5%		-	-	
Hospital teaching status (%)			< 0.001			0.4091
Teaching	72.6%	46.8%		73.1%	74.3%	
Non-teaching	26.4%	52.8%		26.9%	25.7%	
Missing	1.0%	0.5%		-	-	
Hospital bed size (%)			< 0.001			0.0668
Small (1–49 beds)	7.7%	12.2%		7.8%	6.6%	
Medium (50–99 beds)	15.2%	23.7%		15.5%	13.9%	
Large (≥ 100 beds)	76.1%	63.6%		76.6%	79.5%	
Missing	1.0%	0.5%		-	-	

Source: HCUP 2003–2010.

[1] Non-mucormycosis hospitalizations from hospitals with at least one mucormycosis patient were matched 3:1 to mucormycosis hospitalizations using a greedy 8 to 1 digit matching algorithm. This algorithm first matches on 8 digits of propensity score and then subsequently on 7 and so on.

[2] All high-risk groups were mutually exclusive; ordered here by prevalence post-matching. Severely immunocompromised (A) conditions include leukemia/lymphoma/HSCT/BMT, chemotherapy, other immune disorders, solid organ transplant or long-term steroid use. Critically ill (B) conditions include trauma, septicemia, bacteremia, necrotizing fasciitis, intracranial hemorrhage and mechanical ventilation for 96+ hours. Mildly/moderately immunocompromised (C) conditions include ESRD/hemodialysis, lupus, malignant solid tumors, diabetic ketoacidosis, blood disorders, and diabetes. Major surgery or pneumonia (D) includes selected thoracic, cardiac, vascular, abdominal, peripheral, urology, and neurology surgeries, other major procedures, and pneumonia.

[3] Significance was tested for proportions using the Rao-Scott Chi-Square Test. Continuous measures were tested using an independent sample t-test.

[4] Abdominal procedures/surgeries include: ICD-9-CM 43.XX-50.XX, 51.2X, and 52.XX-54.XX, excluding 43.1X, 43.4X, 43.9X, 44.1X, 44.22, 44.43, 44.44, 44.49, 44.91, 45.1X-45.3X, 46.32, 46.32, 46.39, 48.2X, 48.3X, 50.1X, 50.9X, 52.0X, 52.1X, 52.4X, 52.9X, 54.22-54.29, 54.9X.

One-half of the hospitalizations among both cases and non-cases were considered severely immunocompromised (high-risk group A), and one-fifth of cases and non-cases fell into the critically ill (high-risk group B) category. Approximately one quarter of both cases and non-cases were in high-risk groups B and D (major surgery and pneumonia), those considered non-traditional risk factors for mucormycosis. The most common comorbid conditions for cases and non-cases after matching included: hypertension (36.8% and 44.6%, respectively), fluid and electrolyte disorders (42.5% and 27.5%), and diabetes with complications (26.9% and 22.3%).

### Hospital and hospital admission characteristics

Approximately one third of matched hospitalizations were from hospitals in the West and South census regions. Three-quarters of these institutions were teaching hospitals and a slightly higher proportion were large (≥ 100 beds; 76.6% among cases and 79.5% among non-cases). Cases had a higher risk than non-cases of being admitted urgently (26.0% versus 18.2% for non-cases).

### Hospitalization outcomes

Hospitalization outcomes are presented in Tables 2 and 3. Differences in the outcomes between cases and non-cases were larger before matching, but remained substantial and statistically significant after matching. Length of stay was significantly greater among matched cases (mean: 24.5 days; median: 14.0 days) than non-cases (8.0 days; 4.0 days), equating to 16.5 mean excess days of hospitalization among cases (standard error [SE] = 2.2; *P* < 0.001). Inpatient mortality was over five times higher among cases (22.1%) than among matched non-cases (4.4%, mean difference 17.7% [SE 1.3%; *P* < 0.001]). Average total costs varied considerably, with $90,272 (median $51,004) observed among cases and $25,746 (median $12,327) among non-cases. This difference represents $64,526 in mean excess costs for mucormycosis cases (SE = $7,805; *P* < 0.001). Differences between cases and non-cases for all three outcomes persisted over the 8-year timespan of the study, with no clear trend in the magnitude of the difference over time. For example, in-hospital mortality for mucormycosis cases was 25.2% in 2003 and 25.7% in 2009.

**Table 2:** Hospitalization outcomes before and after propensity-score matching

	**Before propensity-score matching**^**1**^					**After propensity-score matching**^**1**^				
**Characteristic**	**Cases**	**Non-Cases**	***p *** **value**^**2**^	**Mean**	**SE**	**Cases**	**Non-Cases**	***p *** **value**^**2**^	**Mean**	**SE**
N (unweighted)	1,120	29,759,178				1,084	3,252			
N (weighted)	5,515	146,295,097				5,346	15,999			
Length of stay (days)										
Mean (SD)	24.5 (32.0)	5.8 (8.3)	< 0.001	18.6	0.4	24.5 (31.9)	8.0 (11.9)	< 0.001	16.5	0.4
Median (IQR)	14 (6–30)	4 (2–7)				14 (6–30)	4 (2–9)			
Mortality (%)	22.2%	3.6%	< 0.001	18.7%	0.6%	22.1%	4.4%	< 0.001	17.7%	0.6%
Discharge type among survivors (%)			< 0.001					< 0.001		
Routine	46.0%	63.3%				46.0%	69.9%			
Short-term hospital	9.6%	2.5%				9.7%	1.8%			
Other transfers^3^	18.5%	19.3%				18.8%	12.3%			
Home health care	24.8%	14.0%				24.3%	14.8%			
Other/unknown	1.1%	0.9%				1.2%	1.1%			
Total Charges										
Mean (SD)	$237,090 ($283,375)	$44,442 ($69,052)	< 0.001	$192,648	$3,816	$237,114 ($285,769)	$71,698 ($114,452)	< 0.001	$165,417	$4,012
Median (IQR)	$128,682	$25,423				$127,171	$33,958			
	($48,025 - $303,348)	($13,423 - $48,779)				($47,653-$301,540)	($17,241 -$74,044)			
Total Costs										
Mean (SD)	$90,107 ($111,760)	$16,582 ($24,509)	< 0.001	$73,525	$1,505	$90,272 ($112,964)	$25,746 ($41,207)	< 0.001	$64,526	$1,579
Median (IQR)	$51,170	$9,943				$51,004	$12,327			
	($17,869 - $112,910)	($5,518 - $18,392)				($17,256 -$112,928)	($6,531 - $27,424)			

Source: HCUP 2003–2010.

[1] Non-mucormycosis hospitalizations from hospitals with at least one mucormycosis patient are matched 3:1 to mucormycosis hospitalizations using a greedy 8 to 1 digit matching algorithm.

[2] Significance was tested for proportions using the Rao-Scott Chi-Square Test. Continuous measures were tested using an independent sample t-test.

[3] Includes skilled nursing facility, intermediate care, and any other type of facility.

Note: Charges and costs reported in 2011 USD.

**Table 3:** Select hospitalization outcomes among the matched cohorts by high-risk group

	**High-risk group**^**1**^			
**Characteristic**	**(A) Severely immunocompromised**	**(B) Critically ill**	**(C) Mildly/moderately immunocompromised**	**(D) Major surgery or Pneumonia**
Mucormycosis Cohort				
N (weighted)	2,623	1,067	1,364	292
Length of stay (days)				
Mean (SD)	25.6 (34.0)	35.6 (37.9)	14 (15.7)	22.5 (30.59)
Median (IQR)	15 (6–32)	25 (10–45)	8 (4–20)	16 (8–24)
Mortality (%)	23.4%	35.3%	9.4%	21.3%
Total Charges				
Mean (SD)	$255,708 ($292,863)	$361,607 ($366,468)	$124,187 ($140,931)	$174,256 ($183,497)
Median (IQR)	$140,867 ($54,693 - $345,646)	$216,589 ($93,922 - $525,537)	$68,281 ($27,062 - $161,126)	$108,226 ($61,543 - $225,011)
Total Costs				
Mean (SD)	$98,365 ($117,047)	$134,082 ($145,047)	$46,923 ($54,558)	$73,748 ($75,906)
Median (IQR)	$56,267 ($22,533 - $122,632)	$85,106 ($33,613 - $173,195)	$24,546 ($10,542 - $62,435)	$45,832 ($20,356 - $92,340)
Non-mucormycosis Cohort				
N (weighted)	7,839	3,488	3,804	867
Length of stay (days)				
Mean (SD)	8.2 (10.67)	10.9 (17.9)	5.3 (6.12)	5.6 (7.8)
Median (IQR)	5 (3–9)	6 (3–13)	4 (2–6)	3 (2–6)
Mortality (%)	3.7%	9.6%	1.5%	1.7%
Total Charges				
Mean (SD)	$77,591 ($125,386)	$96,878 ($136,883)	$38,373 ($48,383)	$62,714 ($77,071)
Median (IQR)	$36,391 ($17,814 - $83,945)	$45,347 ($20,938 - $110,051)	$24,693 ($13,589 - $44,194)	$37,087 ($21,023 - $70,037)
Total Costs				
Mean (SD)	$28,301 ($45,397)	$32,694 ($48,476)	$14,393 ($18,339)	$25,568 ($32,338)
Median (IQR)	$13,229 ($6,618 - $31,261)	$15,488 ($7,820 - $36,035)	$9,094 ($5,168 - $16,155)	$14,343 ($7,534 - $31,615)

Source: HCUP 2003–2010.

[1] All high-risk groups were mutually exclusive. Severely immunocompromised (A) conditions include leukemia/lymphoma/HSCT/BMT, chemotherapy, other immune disorders, solid organ transplant or long-term steroid use. Critically ill (B) conditions include trauma, septicemia, bacteremia, necrotizing fasciitis, intracranial hemorrhage and mechanical ventilation for 96+ hours. Mildly/moderately immunocompromised (C) conditions include ESRD/hemodialysis, lupus, malignant solid tumors, diabetic ketoacidosis, blood disorders, and diabetes. Major surgery or pneumonia (D) includes selected thoracic, cardiac, vascular, abdominal, peripheral, urology, and neurology surgeries, other major procedures, and pneumonia.

Note: Charges and costs reported in 2011 USD.

Critically ill patients (high-risk group B) demonstrated the largest differences in the outcomes between matched cases and non-cases (excess mortality: 25.7%; mean excess LOS: 24.7 days, [median difference: 19 days]); mean excess costs: $101,388, [median difference: $69,618]), followed by the severely immunocompromised patients (high-risk group A; 19.7%; 17.4 days [10 days]; $70,064 [$43,038]). Patients considered only mildly or moderately immunocompromised (high-risk group C) or those with major surgery or pneumonia (high-risk group D) exhibited smaller differences in these outcomes (C: 7.9%, 8.7 days [4 days], $32,530 [$15,452]; D: 19.5%, 16.9 days [13 days], $48,181 [$31,489]).

## Discussion

We confirm that mucormycosis remains a rare infection, present in less than 0.01% of all US hospitalizations between 2003 and 2010. Some degree of immune compromise, identified in three-quarters of admissions with this diagnosis, continues to represent the most common risk factor for this infection. The remainder, however, carried coexisting conditions less traditionally recognized as associated with mucormycosis, such as critical illness, pneumonia, and major surgery. Of the admissions over the eight years examined carrying a mucormycosis ICD-9-CM code, only 6% failed to fall into one of the four high-risk groups. Propensity-score matched analysis revealed a dramatic rise in hospital mortality (mean excess 17.7% [SE 1.3%]), LOS prolongation (mean excess days 16.5 [SE 2.2]) and costs (mean excess costs $64,526 [SE $7,805]) associated with a diagnosis mucormycosis.

Prior studies examining outcomes among patients with mucormycosis have been limited by small numbers of patients with mucormycosis examined as a subset of those with IFIs. For example, a recent single center study by Dodds and colleagues [13] compared 200 patients with an IFI diagnosis (including 4 mucormycosis cases and also cases of aspergillosis, cryptococcosis, and invasive candidiasis) to 200 matched controls without IFIs. In this study, IFI diagnosis imparted a mean excess of 7.4 days in hospital LOS along with approximately $37,000 (2011 USD) in excess cost.

Menzin and colleagues [15] examined the economic burden of IFIs using a single year (2004) of the HCUP –NIS. In this matched cohort study of nearly 12,000 IFI hospitalizations, only 119 had a mucormycosis ICD-9-CM code. Those with mucormycosis suffered a 12% higher absolute risk of hospital mortality and a 10.7-day excess in the hospital LOS, corresponding to approximately $41,000 (2011 USD) in excess costs. Our study adds power and precision to these results, given our focus specifically on mucormycosis and the large number of cases. Data from this study generally confirm the findings from both of these earlier reports [13,15]. Compared to the analysis by Dodds, our finding of an excess mean LOS of 16.5 days and costs of $64,526 emphasizes the disproportionate financial impact of mucormycosis. Our findings also add to and build on those of Menzin et al., in that our results not only draw on the more recent data from HCUP-NIS and on a much larger cohort of patients with mucormycosis, but reflect the efforts of a more rigorous matching algorithm. This helps to assure greater internal validity. Moreover, specific to mucormycosis, our clinical and economic results are highly generalizable within the US as they are derived from a representative sample of US acute care institutions.

Although generalizable and internally valid, our study needs to be interpreted with some caution. One concern stems from the similarity of our mortality data to that observed in a specific population of patients with mucormycosis. Hammond and colleagues [1] studied 30 adult hematopoietic stem cell transplant or hematologic malignancy patients with mucormycosis and reported a 6-week mortality rate of 20%. Our aggregate mortality rate was similar to what is reported by Hammond and colleagues. However, our stratified mortality data add further granularity by untangling the heterogeneity in this outcome as a function of the specific risk factors for mucormycosis. Indeed, the mortality rate among those with severe immune compromise in our study mirrors that in the analysis by Hammond et al. In contrast, we found hospital mortality among those with mild immune compromise to be substantially lower than among the severely immunocompromised. At the same time, among those who were critically ill mortality was even higher than among those with severe immune compromise.

Our study is subject to a number of additional limitations. First, it was cross-sectional, and thus did not allow us to explore hospital events in relation to the onset of mucormycosis. For this reason it is likely that at least some of the excess days and costs seen in mucormycosis group were incurred prior to the onset of mucormycosis. Therefore, we were unable to assess the degree to which this burden was specifically attributable to mucormycosis. The cross-sectional nature of the analysis (and database) also precluded the ability to factor the timing of concomitant diagnoses within the admission into the high-risk categorization process. Due to variability in data availability across years, not all hospitalization characteristics were included as predictors in the logistic regression to estimate propensity scores. Specifically, admission source (approximately 30% missing) and admission type (approximately 20% missing) were excluded.

Furthermore, we relied on diagnosis codes to identify cases of mucormycosis. This predisposes our case identification to potential misclassification, including inadvertent exclusion of “probable” and “possible” mucormycosis cases. However, if this misclassification is non-differential, then the magnitude of group differences found in our study is actually likely to be biased toward the null. In other words, if such non-differential misclassification exists, then the actual between-group differences in the outcomes are potentially even greater than what we computed. Lack of clinical data also limited our ability to adjust for confounding clinical characteristics when assessing excess costs, such as the site of the mucormycosis infection. In addition, the cost analysis did not include costs of physician services, patient out-of-pocket expenses, indirect costs, costs post-discharge, or costs in the outpatient setting; therefore, this study may have underestimated the true burden of mucormycosis from a societal perspective.

## Conclusions

In summary, mucormycosis remains a rare but deadly infection among hospitalized patients. In addition to its clinical burden, it is associated with a vast increase in hospital resource use as measured by both LOS and costs. Finally, approximately one quarter of all admissions afflicted with mucormycosis have non-traditional risk factors for this infection such as critical illness, pneumonia, and major surgery. Although rare, by virtue of its dismal outcomes, mucormycosis demands more concentrated attention from the research and clinical communities.

## Abbreviations

AHRQ: Agency for Healthcare Research and Quality; HCUP: Healthcare Cost and Utilization Project; ICD-9-CM: International Classification of Diseases, 9th Revision, Clinical Modification; IFI: Invasive fungal infection; IQR: Interquartile range; LOS: Length of stay; NIS: Nationwide Inpatient Sample; PS: Propensity score; SE: Standard error; SD: Standard deviation; TRANSNET: Transplant Associated Infections Surveillance Network; US: United States; USD: United States Dollars.

## Competing interests

HH, VF, and JM are employees of Boston Health Economics, Inc. and were funded by Astellas Scientific and Medical Affairs, Inc. for this study. PC is an employee of, and MZ and AS are consultants to the sponsor. MZ is also a consultant to Cubist Pharmaceuticals, Pfizer, and Optimer Pharmaceuticals. AS is also a consultant to Cubist Pharmaceuticals, Forest Pharmaceuticals, Inc., Bayer, Pfizer, Theravance, and Trius Therapeutics. The authors have no other financial or non-financial competing interests to disclose.

## Authors’ contributions

MZ, AS and JM were involved in the conception and design of the study, the interpretation of data, and revised the manuscript critically. HH and VF contributed to the design of the study, the acquisition of data, the analysis and interpretation of data, and drafting the manuscript. PC contributed to the design of the study, the interpretation of data, and the critical revision of the manuscript. All authors have read and approved the final manuscript.

## Pre-publication history

The pre-publication history for this paper can be accessed here:

http://www.biomedcentral.com/1471-2334/14/310/prepub
